# A Novel Strain *Burkholderia theae* GS2Y Exhibits Strong Biocontrol Potential Against Fungal Diseases in Tea Plants (*Camellia sinensis*)

**DOI:** 10.3390/cells13211768

**Published:** 2024-10-25

**Authors:** Yijie Dong, Xing Wang, Guang-Da Feng, Qing Yao, Honghui Zhu

**Affiliations:** 1 Key Laboratory of Agricultural Microbiomics and Precision Application (MARA), Guangdong Provincial Key Laboratory of Microbial Culture Collection and Application, Key Laboratory of Agricultural Microbiome (MARA), State Key Laboratory of Applied Microbiology Southern China, Guangdong Microbial Culture Collection Center (GDMCC), Institute of Microbiology, Guangdong Academy of Sciences, Guangzhou 510070, China; dongyj@gdim.cn (Y.D.); wangxingx7@163.com (X.W.); fenggd@gdim.cn (G.-D.F.); 2 Guangdong Province Key Laboratory of Microbial Signals and Disease Control, Guangdong Engineering Research Center for Litchi, College of Horticulture, South China Agricultural University, Guangzhou 510642, China

**Keywords:** *Burkholderia theae*, tea pathogenic fungi, biocontrol, tea plants (*Camellia sinensis*)

## Abstract

Background: Tea plants (*Camellia sinensis*) are widely cultivated cash crops. However, fungal diseases lead to significant reductions in both the yield and quality of tea. Therefore, searching for economical, eco-friendly, and efficient biological control measures is crucial for protecting tea plants from pathogenic fungi. Methods: The confrontation assays were performed to identify the antagonistic bacteria against tea pathogenic fungi and evaluate the antifungal activity of these bacteria. Results: Here, three tea pathogenic fungi were identified: *Colletotrichum siamense* HT-1, *Diaporthe phaseolorum* HT-3, and *Fusarium fujikuroi* HT-4. Notably, *D. phaseolorum* was the first to be reported in tea plants in China. Some tea pathogenic fungi showed a high relative abundance, suggesting a potential disease risk in tea plantations. Strain GS2Y, isolated from tea rhizosphere soil, exhibited strong antifungal activity against tea pathogenic fungi and represented a novel species within the genus *Burkholderia*, designated as *Burkholderia theae*. GS2Y could directly inhibit tea pathogenic fungi by disrupting the cellular structures and protect tea plants from fungal diseases caused by *C. siamense* HT-1 and *D. phaseolorum* HT-3. Conclusions: *B. theae* GS2Y might function as a potentially valuable resource for biocontrol agents, laying the foundation for the development of strategies to manage fungal diseases in tea plants.

## 1. Introduction

Tea (*Camellia sinensis*) is one of the most important economic crops and is widely cultivated in tropical and subtropical regions, including Asia, Africa, and South America [1,2,3]. Tea contains various chemical compounds that are beneficial to human health and can effectively reduce the risk of diseases [4,5]. In 2021, the total area of tea plantations in China was approximately 3.26 million hectares, with an output value of USD 45.4 billion, making it the largest producer worldwide [6,7]. As perennial woody plants, tea plants frequently face multiple biotic and abiotic threats during their long life. Among the various biotic threats, fungal diseases lead to a significant decrease in both the yield and quality of tea [3]. Previous studies have shown that tea brown blight was the most damaging and common foliar disease of the tea plant in China and caused a substantial 30–50% reduction in tea yield [8,9]. Over 500 fungal pathogenic species have been reported to cause diseases in tea plants in China [10].

Fungal pathogens can infect all parts of the tea plant, including the foliage, stems, and roots. Foliar diseases directly impact the harvest, while stem and root diseases affect the survival of tea plants [11]. Among foliar diseases, blister blight, gray blight, and brown blight are particularly significant, as they adversely impact the buds and the two youngest leaves, resulting in a loss of harvestable shoots [3,12,13,14]. Blister blight caused by *Exobasidium vexans Massee* is one of the most serious diseases of tea plants, one which occurs in almost all tea-growing countries [15]. In Southwest China, the incidence of tea blister blight can reach 40–50% and even 90% in severe cases [14]. Gray blight disease, caused by several *Pestalotiopsis*-like species, initially manifests as brown concentric spots in the center of the leaf. These spots later turn gray with brown margins and spread throughout the entire leaf, ultimately damaging young developing shoots [16]. Gray blight has led to economic losses of up to 50% in Hubei Province, China [17]. *Colletotrichum* species are the primary pathogens responsible for brown blight, including *C. camelliae*, *C. acutatum*, *C. siamense*, *C. fructicola,* and *C. jiangxiense* [18,19,20,21,22]. Brown blight disease manifests as cloudy symptoms characterized by large, gray-white lesions on tea leaves. Infected plants often exhibit dieback of tender branches and defoliation, and in some sensitive varieties, the entire plant may even succumb to the disease. The incidence of brown blight caused by *C. fructicola* was estimated to range from 30% to 50%, leading to significant defoliation in Guangdong Province, China [23]. For an extended period, chemical control served as the primary method for managing fungal diseases in tea plants. However, there are a limited number of fungicides registered for use against tea plant diseases, and excessive application of synthetic fungicides has led to a series of issues such as pathogen resistance, fungicide residues, and environmental pollution [24]. Consequently, a sustainable, environmentally friendly, and modern biological control technology is urgently required to replace traditional chemical control in tea plantations.

Rhizosphere microbes play an important role in enhancing plant health and improving plant resistance to both biotic and abiotic stresses [25]. Additionally, these microbes serve as primary sources of microbial biocontrol agents [26]. Some microorganisms, such as *Actinomycetes*, *Bacillus*, *Pseudomonas*, and *Trichoderma*, have been employed to control fungal diseases of tea plants [27,28]. For instance, dinactin derived from *Streptomyces badius* gz-8 exhibited strong inhibitory effects against rubber anthracnose caused by *C. gloeosporioides* [29]. Macrolactin derivatives isolated from *Bacillus subtilis* B5 showed antifungal activities against the tea pathogenic fungi *P. theae* and *C. gloeosporioides* [30]. Furthermore, *P. aeruginosa* RTE4, isolated from tea rhizosphere soil, significantly inhibited two foliar fungal pathogens of tea, namely, *C. invisium* and *Fusarium solani* [31]. *Trichoderma reesei* TRPATH01 enhanced the resistance of tea plants to gray blight by upregulating the expression of defensive enzymes, including polyphenol oxidase, peroxidase, phenylalanine ammonia lyase, β-1,3-glucanase, and chitinase [32].

The *Burkholderia* genus is ubiquitous in soil and commonly found in the rhizosphere [33,34]. Given that the genome sizes of the *Burkholderia* genus range from 5.23 Mb in *B. mallei* SAVP to 10.35 Mb in *B. contaminans* LMG 23361, this variability indicates the genus’s capacity to colonize diverse and complex environments [35]. Despite being widely recognized for their pathogenic effects on humans, animals, and plants, numerous species within this genus also exhibit beneficial properties for plants [33]. These species can establish a beneficial relationship with plants by either promoting plant growth or inhibiting phytopathogens with the production of antimicrobial compounds, such as pyrrolnitrin, burkholdine, and cepacin [34]. Previous studies have shown that *Burkholderia* and *Pseudomonas* are the dominant genera in the rhizosphere soil of tea plants, while the abundance of plant growth-promoting rhizobacteria, especially *Bacillus*, *Prevotella*, and *Sphingomonas*, significantly decreases with prolonged cultivation [36,37]. *B. pyrrocinia* P10, isolated from the rhizosphere region of tea, can solubilize phosphorus compounds, produce indole-3-acetic acid, and secrete siderophores [38]. A previous study has demonstrated that the volatile organic compounds (VOCs) produced by *B. pyrrocinia* strain JK-SH007 inhibit three pathogens causing poplar canker (*Cytospora chrysosperma*, *Phomopsis macrospora*, and *Fusicoccum aesculi*) [39].

In this study, tea pathogenic fungi were identified in tea plantations located in Heshan City, Guangdong Province, China. An antagonistic strain, designated GS2Y, was isolated from the rhizosphere soil of tea plants, which exhibited strong antifungal activity against tea pathogenic fungi. To gain a deeper understanding of strain GS2Y, a series of experiments was performed for taxonomic characterization through morphological, phylogenetic, and physiobiochemical assays. We also evaluated the biocontrol potential of strain GS2Y against tea fungal pathogens. These findings provide a foundation for the development of potential biocontrol agents for managing fungal diseases in tea plants.

## 2. Materials and Methods

### 2.1. Isolation of Tea Pathogenic Fungi and Pathogenicity Tests

Diseased leaves, which were collected from tea plantations in Heshan City, were disinfected on the surface using 70% ethanol for 1 min, followed by treatment with a 0.5% sodium hypochlorite solution (Mreda, Beijing, China) for 2 min. Subsequently, the leaves were rinsed three times with a substantial volume of sterilized water to thoroughly eliminate any remaining sterilizing agent. The diseased tissues adjacent to the asymptomatic regions were cut into small pieces (5 mm^2^) and subsequently placed on potato dextrose agar (PDA) supplemented with chloramphenicol (20 µg mL^−1^) (Aladdin, Shanghai, China). The plates were incubated at 25 °C until hyphae emerged from the necrotic tissue. Single hyphal tips were then excised and transferred onto PDA (BD Difco) for further incubation at 25 °C [13]. Pathogenicity tests using mycelia were conducted as previously described [40]. Fresh tea leaves were punctured with a fine needle at the midpoint of each leaf. A 6 mm diameter mycelial disc was excised from a 5-day-old culture of the tea pathogenic fungus and placed onto the tea leaf, ensuring that the hyphal side was in contact with the leaf surface. The inoculated leaves were then placed in a sterile Petri dish with two pieces of moistened filter paper. Subsequently, the plates were incubated in an illuminated chamber at 25 °C for one week, alternating between 12 h of light and 12 h of darkness. PDA discs were included in parallel as negative controls. Five leaves were used for each treatment, and symptoms were observed daily. At least three biological replicates were performed.

### 2.2. Isolation of Antagonistic Strains

To obtain the antagonistic strains of tea fungal pathogens, the cultivable bacteria were isolated from the rhizosphere soil and leaves of tea plants (*Camellia sinensis*). Briefly, 5 g soil was suspended into 95 mL sterile water, and the mixture was shaken at 30 °C for 30 min. The suspension was spread on R2A agar (Hope, Qingdao, China) using the standard dilution method. The 16S rRNA gene of bacterial colonies was amplified and sequenced with the bacterial universal primer set 27F/1492R by GENEWIZ (Suzhou, China) [41]. The 16S rRNA gene sequence similarities of cultivable bacteria and close type strains were obtained using the EzBioCloud sever (https://www.ezbiocloud.net/, accessed on 1 September 2024) [42]. Subsequently, the antifungal activity of these strains was assessed on an R2A plates using the plate confrontation experiment and *D. phaseolorum* HT-3 as the indicators. At least three biological replicates were performed.

### 2.3. Identification of Strain GS2Y

Genome sequencing of strain GS2Y was performed using the Illumina HiSeq platform by Majorbio Co., Ltd. (Shanghai, China). The sequences were assembled with SPAdes software version 3.15.0 [43]. Phylogenomic analyses were conducted using UBCG (Up-To-Date Bacterial Core Gene) [44] and TYGS (Type (Strain) Genome Server) (https://tygs.dsmz.de/, accessed on 30 August 2024) [45], both utilizing default settings. The average nucleotide identity (ANI) and digital DNA-DNA hybridization (dDDH) relatedness between strain GS2Y and type strains were calculated using the ANI Calculator (http://www.ezbiocloud.net/tools/ani, accessed on 30 August 2024) [46] and the Genome-to-Genome Distance Calculator (GGDC 3.0) (http://ggdc.dsmz.de/distcalc2.php, accessed on 30 August 2024) [47], respectively. The API 20NE was used to determine the corresponding properties according to the manufacturer’s instructions. Cellular fatty acids were extracted and identified with gas chromatography (model 7890A, Agilent, Santa Clara, CA, USA) using the Microbial Identification System package with the Sherlock MIDI 6.1 and the Sherlock Aerobic Bacterial Database (TSBA 6.1). Respiratory quinones were extracted and analyzed using an HPLC (Shimadzu LC-20A, Kyoto, Japan) as described previously [48].

### 2.4. Assessment of Antifungal Activity of Strain GS2Y

The antifungal activity of strain GS2Y was evaluated using the plate confrontation experiment. Briefly, 6 mm diameter mycelial discs from 5-day tea pathogenic fungi HT-1 or HT-3 were inoculated onto PDA plates. Overnight cultures of strain GS2Y were grown in LB medium until they reached the mid-exponential phase. The cells were harvested, washed twice with PBS buffer (137 mM NaCl, 2.7 mM KCl, 10 mM Na_2_HPO_4_, 1.8 mM KH_2_PO_4_, pH 7.2), and then re-suspended in PBS to an optical density (OD_600nm_) of 1.0. A 3.0 μL aliquot of the strain GS2Y suspension was applied in a straight line on all four sides of the mycelial disc, maintaining a distance of 20 mm from the disc. The plates were incubated at 30 °C for 5 days. Each treatment included at least five biological replicates, and the assay was conducted in triplicate. To evaluate the antifungal activity of the GS2Y strain fermentation supernatant and metabolic crude extract, growth inhibition was performed using the tea pathogenic fungi HT-1, HT-3, and HT-4 as indicators. In brief, overnight cultures of strain GS2Y were re-suspended in LB liquid medium to an OD_600_ of 0.1 and cultured at 30 °C for 48 h. The fermentation supernatant was collected through centrifugation and filtered using a 0.22 μm filter. Subsequently, the fermentation supernatant of strain GS2Y was mixed with PDA medium to achieve a final concentration of 10%, which was then poured into a Petri dish. The macroporous resin XAD-16 (Yuanye, Shanghai, China) was used for preparation of antifungal crude extracts [49]. The resin XAD-16 was added to the harvested fermentation supernatant of strain GS2Y and separated from the fermentation supernatant using filtration and then extracted twice with methanol (1 × 100 mL and 1 × 50 mL). The combined extract was concentrated to dryness using a rotary evaporator. The residue was re-dissolved in deionized water and subsequently filtered through a 0.22 μm pore size syringe microfilter to obtain the metabolic crude extract. The metabolic crude extract was added to the PDA medium to achieve a final concentration of 1.25 mg mL^−1^. A 6 mm diameter mycelial disc, obtained from HT-1, HT-3, and HT-4 grown on PDA for five days, was placed at the center of the plate and incubated at 30 °C. After five days, the diameter of the growth inhibition zone was measured. At least three biological replicates were performed.

### 2.5. Microscopic Observations

To elucidate the mechanism by which GS2Y inhibits pathogenic fungi in tea plants, the hyphal morphology was observed using a fluorescence microscope and scanning electron microscopy (SEM). The 10% fermentation supernatant obtained from strain GS2Y was co-cultured with HT-1 and HT-3 for two days, the hyphal morphology was observed using a fluorescence microscope (ZEISS, Axio scope 5). For field emission scanning electron microscopy (FESEM), the samples were fixed overnight at 4 °C in a 3% glutaraldehyde solution. After overnight incubation, fixation solution was removed carefully. Subsequently, the samples were washed once with 0.1 M, pH 7.0 phosphate buffer for 15 min. Later, the samples were subjected to dehydration treatment with concentration gradients (30%, 50%, 70%, 90%, and 100%) of ethanol solution and treated at each concentration for 15 min. Finally, the ethanol was removed, and the samples were observed using SEM [50].

### 2.6. Disease Control

To evaluate the antifungal activity of GS2Y in tea leaves, sixty fresh leaves were collected. Each leaf was punctured with a fine needle at the midpoint and positioned on two pieces of moist filter paper within a sterile Petri dish. These leaves were categorized into three distinct groups: the first group was treated with a 10% fermentation supernatant, the second group was exposed to a metabolic crude extract at a concentration of 1.25 mg mL^−1^, and the third group was treated with sterilized water as the control group. A single piece of hypha from strain HT-1 or HT-3 was then placed onto each leaf, ensuring that the hyphal side was in contact with the leaf surface. Subsequently, the plates were incubated in an illuminated chamber for one week at 25 °C, alternating between 12 h of light and 12 h of darkness, to assess the capability of GS2Y in combating tea fungal diseases. Each treatment group comprised a minimum of five leaves, ensuring a robust evaluation of the antifungal efficacy. At least three biological replicates were performed.

### 2.7. Statistical Analysis

The measurements of the antifungal activity of strain GS2Y were derived from average values obtained from a minimum of three independent experiments. Statistical analysis was performed using GraphPad Prism software 8.0.2, and statistical significance was determined with a value of *p* < 0.05.

## 3. Results

### 3.1. Identification and Characterization of Tea Pathogenic Fungi

**Figure 1:**
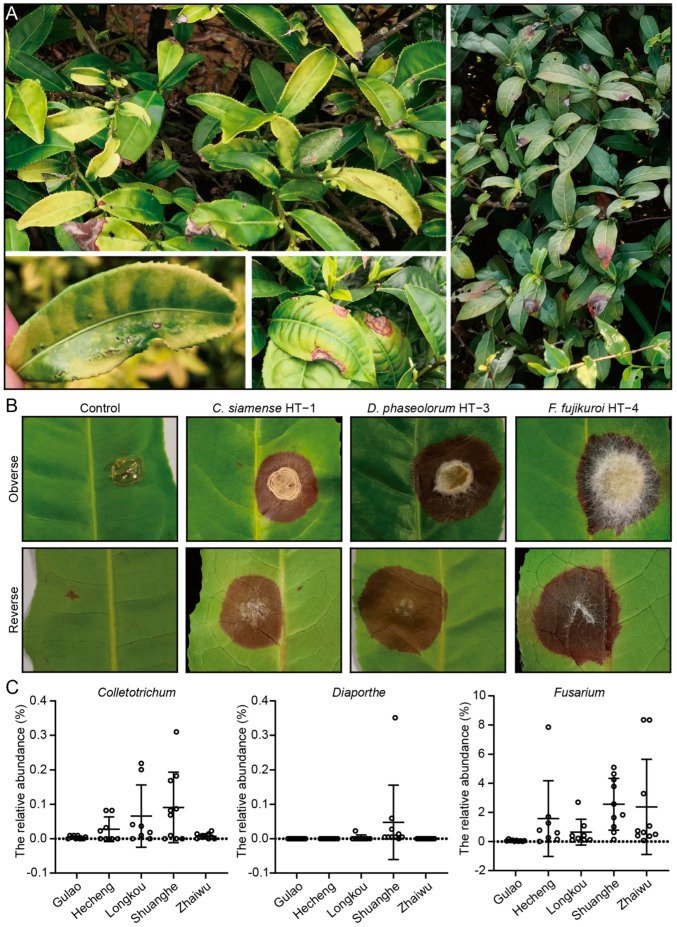
Investigation and identification of pathogenic fungi from tea plantations in Heshan, Southern China. (**A**) Typical symptoms were found in leaves of tree plants from tea plantations in Heshan. (**B**) Pathogenicity tests of the potential tea pathogenic fungi. The 6 mm diameter mycelial discs of strains HT-1, HT-3, and HT-4 were incubated with fresh tea leaves, which were scratched using a fine needle at the leaf midpoint. The leaves were placed in a sterile Petri dish and incubated in an illuminated chamber at 25 °C for one week. Subsequently, the plates were alternated between 12 h of light and 12 h of darkness. The symptoms were recorded using a camera. (**C**) The relative abundance of the genera *Colletotrichum*, *Diaporthe*, and *Fusarium* was investigated based on the results of high-throughput sequencing. The circles represent the data points in the graph. Gulao, Hecheng, Longkou, Shuanghe, and Zhaiwu represent samples from tea plantations in Gulao town, Hecheng town, Longkou town, Shuanghe town, and Zhaiwu town, respectively.

Tea is primarily produced from the young shoots of tea plants; consequently, effective management of foliar diseases is crucial for ensuring both the yield and the quality of the tea [3]. In our previous study, the soil fungal community structure of tea plantations was investigated in Heshan, Southern China [51]. The results indicated a significant presence of infected leaves in the tea plants (Figure 1A). Three potential pathogenic fungi were isolated from the infected leaves, designated as HT-1, HT-3, and HT-4 (Figure S1). Based on a sequence comparison of the internal transcribed spacer (ITS) region, strains HT-1, HT-3, and HT-4 belonged to *Colletotrichum siamense*, *Diaporthe phaseolorum*, and *Fusarium fujikuroi*, respectively. To confirm the infected capacity of these strains, the pathogenicity tests were performed. The results show that strains HT-1, HT-3, and HT-4 caused symptoms on wounded inoculated sites on tea leaves (Figure 1B), indicating that these strains might enter tea leaves via wounds. Based on both DNA sequence data and morphological evidence, the fungi isolated from the wound sites fulfilled Koch’s postulates, indicating that *C. siamense* HT-1, *D. phaseolorum* HT-3, and *F. fujikuroi* HT-4 were the pathogens of tea plants. To our knowledge, this is the first report of *D. phaseolorum* causing foliar disease on tea plants (*Camellia sinensis*) in China. Furthermore, high-throughput sequencing was employed to investigate potential tea pathogenic fungi in tea plantations. The results showed a high abundance of pathogenic fungi in the rhizosphere soil of tea plants, including the genera *Colletotrichum*, *Diaporthe*, *Fusarium*, *Pseudopestalotiopsis*, *Alternaria*, and *Clonostachys* (Figure 1C and Figure S2). Previous studies have confirmed that pathogens from these genera cause destructive fungal diseases in tea plants [23,52,53,54,55,56]. These results suggest that *C. siamense* HT-1, *D. phaseolorum* HT-3, and *F. fujikuroi* HT-4 are pathogenic to tea plants, indicating a potential disease risk in the tea plantations of Heshan in Southern China.

### 3.2. Isolation of Antagonistic Bacteria Against Tea Pathogenic Fungi

**Figure 2:**
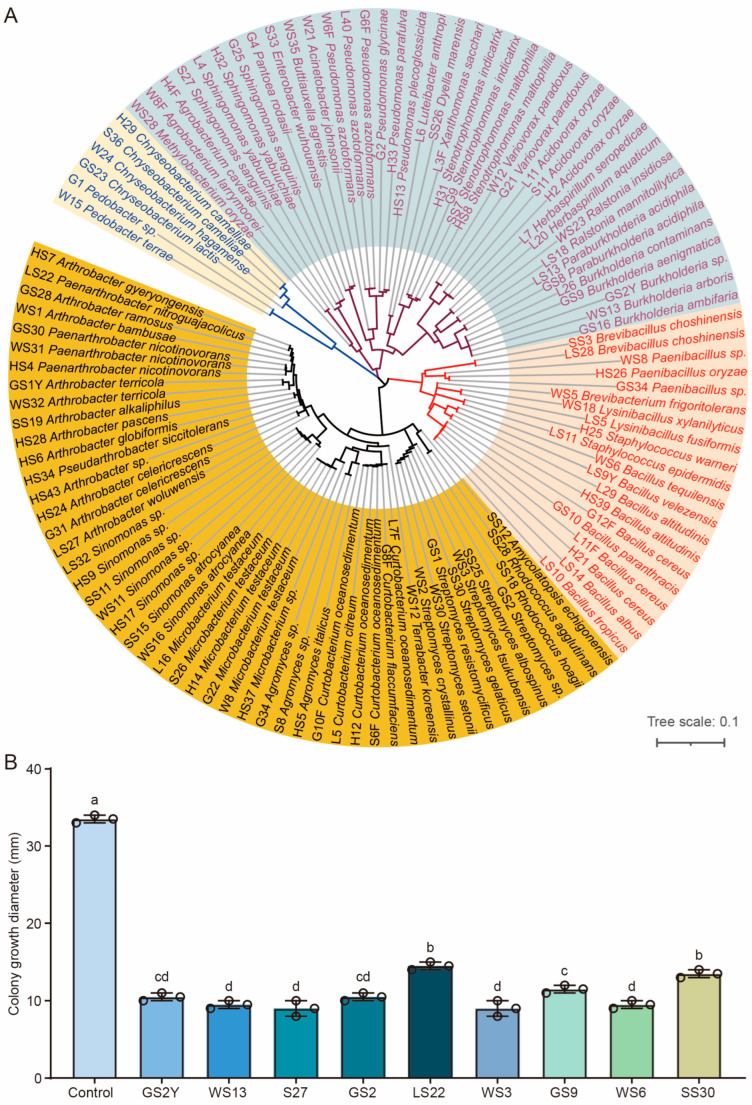
Isolation of cultivable bacteria and screening of antagonistic strains from the rhizosphere soil and leaves of tea plants. (**A**) Phylogenetic analysis of cultivable bacteria isolated from the rhizosphere soil and leaves of tea plants. The neighbor-joining tree was constructed based on 16S rRNA gene sequences. Scale bar: 0.01 nucleotide substitution rate (*Knuc*) units. (**B**) Diameters of fungal colonies were measured following confrontation culture. The circles represent the data points in the graph. The letters (a to d) above the columns indicate significant differences at *p* < 0.05, as determined using an unpaired two-tailed Student’s *t*-test analysis.

Tea plants infected with fungal pathogens result in significant losses in both yield and quality [3,15]. Therefore, it is crucial to investigate potential beneficial microorganisms in the tea rhizosphere for the effective management of tea fungal diseases [57]. To identify the antagonistic bacteria that can combat pathogenic fungi affecting tea plants, the cultivable bacteria were isolated from the rhizosphere soil and leaves of tea plants. A total of 116 isolates were obtained, and the corresponding 16S rRNA gene sequences were obtained using PCR amplification followed by sequencing. The taxonomic status of these isolates was determined by conducting a comparison of 16S rRNA gene sequences using the EzBioCloud database (Table S1). These isolates were classified into four phyla: *Actinobacteria*, *Proteobacteria*, *Firmicutes*, and *Bacteroidetes* (Figure 2A). Among them, the predominant genera included *Arthrobacter*, *Bacillus*, *Streptomyces*, *Sinomonas*, *Pseudomonas*, *Microbacterium*, *Curtobacterium*, and *Burkholderia* (Figure 2A). Furthermore, the antagonistic activity of these bacteria against *D. phaseolorum* HT-3 was analyzed using the plate confrontation experiment. The results demonstrated that nine strains exhibited strong antagonistic activity: three strains of *Burkholderia* genus (strains GS9, WS13, and GS2Y), three strains of *Streptomyces* genus (strains SS30, GS2, and WS3), one strain of *Bacillus* genus (strain WS6), one strain of *Paenarthrobacter* genus (strain LS22), and one strain of *Sphingomonas* genus (strain S27) (Figure 2B). These strains might serve as potential candidates for the development of biocontrol agents.

### 3.3. Morphological, Physiological, and Biochemical Characteristics of Strain GS2Y

**Figure 3:**
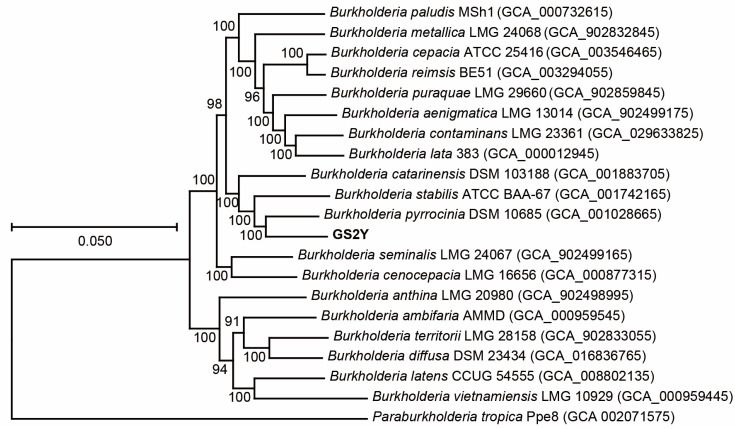
The maximum-likelihood phylogenetic tree based on core genes showing the phylogenetic positions of strain GS2Y and related reference strains. *Paraburkholderia tropica* Ppe8 was used as the outgroup. Bootstrap values greater than 50% are shown at branch points. Bar: 0.10 nucleotide substitution rate (*Knuc*) units.

*Burkholderia* species have been effectively employed as commercial biopesticides owing to their fungicidal properties and protective effects on plants [34,58]. Based on the results of the confrontation experiment, the antagonistic strain GS2Y was further identified with the morphological, phylogenetic, and physiobiochemical assays. The results showed that the genomic DNA G + C contents of strain GS2Y were 66.5% (Table S2). Phylogenomic analysis indicated that strain GS2Y and *Burkholderia pyrrocinia* DSM 10685 formed an independent branch (Figure 3). The average nucleotide identity (ANI) and digital DNA-DNA hybridization (dDDH) values between strains GS2Y and *B. pyrrocinia* DSM 10685 were 52.2% and 93.9%, respectively (Table S3). The dDDH and ANI values were far below the recognized thresholds of 70% dDDH [59] and 95–96% ANI [60] for species delineation, indicating that strain GS2Y might be a member of the putative new species of the genus *Burkholderia*. Colonies of strain GS2Y grown on LB agar were light yellow, circular, smooth, and convex (Figure S3A,B). Cells were rod-shaped, measuring 0.6–0.8 µm in width and 1.8–2.4 µm in length, with flagella (Figure S3C). The reduction of nitrate and utilization of D-maltose of strain GS2Y were positive, but they were negative for related reference strains (Table S4). Strain GS2Y could solubilize organic and inorganic phosphorus (Table S4), indicating that it might have a plant growth promotion function. The predominant ubiquinone of strain GS2Y was Q-8. The predominant cellular fatty acids of strain GS2Y (>10.0%) were C_16:00_ (23.95%), C_17:0_ cyclo (21.53%), and C_19:0_ cyclo *w*8*c* (22.18%) (Table S5). These results demonstrate that strain GS2Y is considered to represent a novel species of the genus *Burkholderia*, for which the name *Burkholderia theae* is proposed.

### 3.4. GS2Y Can Directly Affect Tea Pathogenic Fungi

**Figure 4:**
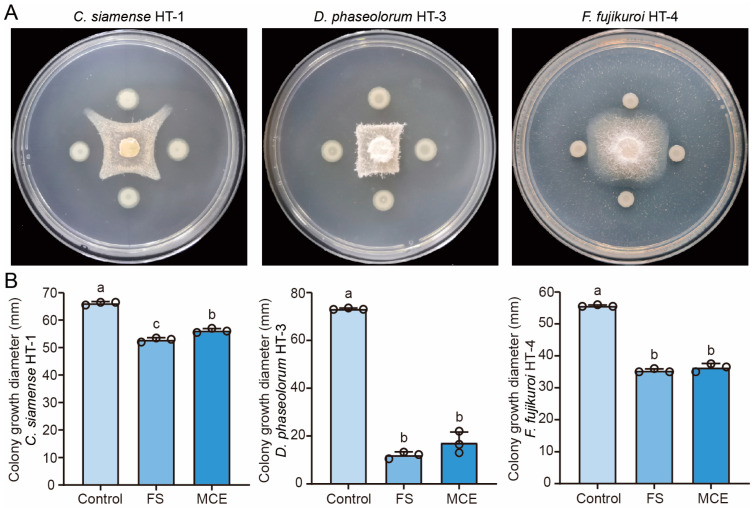
Antifungal activity of strain GS2Y on tea pathogenic fungi. (**A**) Confrontation culture assays were performed to detect strain GS2Y against *C. siamense* HT-1, *D. phaseolorum* HT-3, and *F. fujikuroi* HT-4. Mycelial discs of HT-1, HT-3, and HT-4 were placed in the center of PDA plates, and a suspension of strain GS2Y was streaked in a straight line around all four sides. The plates were incubated at 30 °C for 5 days prior to photography. Each treatment included at least three biological replicates. (**B**) Growth inhibition tests were conducted to measure the antifungal activity of fermentation supernatant (FS) and metabolic crude extract (MCE) derived from strain GS2Y. A 6 mm diameter mycelial disc of HT-1, HT-3, or HT-4 grown on PDA for five days was placed on the PDA plates containing 10% fermentation supernatant or a final concentration of 1.25 mg mL^−1^ of metabolic crude extract. The plates were incubated at 30 °C for five days, and the diameter of the growth inhibition zone was measured. The circles represent the data points in the graph. The letters (a to c) above the columns indicate significant differences at *p* < 0.05, as determined using an unpaired two-tailed Student’s *t*-test analysis.

To assess the antifungal activity of strain GS2Y against tea pathogenic fungi, the confrontation experiment was performed using *C. siamense* HT-1, *D. phaseolorum* HT-3, and *F. fujikuroi* HT-4 as the indicators. Based on the results, strain GS2Y exhibited strong antagonistic activity against *C. siamense* HT-1, *D. phaseolorum* HT-3, and *F. fujikuroi* HT-4, especially HT-1 and HT-3 (Figure 4A). In addition, the results suggested that strain GS2Y also showed significant antagonistic activity against plant pathogenic fungi, such as *Phyllosticta captalensis* and *Gloeosporium musarum* (Figure S4A,B). The antifungal activity of fermentation supernatant and metabolic crude extract derived from strain GS2Y against *C. siamense* HT-1, *D. phaseolorum* HT-3, and *F. fujikuroi* HT-4 was further measured based on growth inhibition. Our results demonstrated that fermentation supernatant and metabolic crude extract significantly inhibited the growth of tea pathogenic fungi *C. siamense* HT-1, *D. phaseolorum* HT-3, and *F. fujikuroi* HT-4, particularly against *D. phaseolorum* HT-3 (Figure 4B). To determine the minimum inhibitory concentrations of metabolic crude extract, 3.0, 4.0, and 5.0 mg mL^−1^ concentrations of metabolic crude extract were used for detecting the antifungal activity against three tea pathogenic fungi. The results demonstrated that the minimum inhibitory concentrations of metabolic crude extract against *C. siamense* HT-1, *D. phaseolorum* HT-3, and *F. fujikuroi* HT-4 were 4.0, 3.0, and 5.0 mg mL^−1^, respectively (Figures S5–S7). Overall, these findings indicate that strain GS2Y has a wide spectrum of inhibitory actions against plant pathogens.

### 3.5. GS2Y Disturbs the Cellular Structure of Tea Pathogenic Fungi

**Figure 5:**
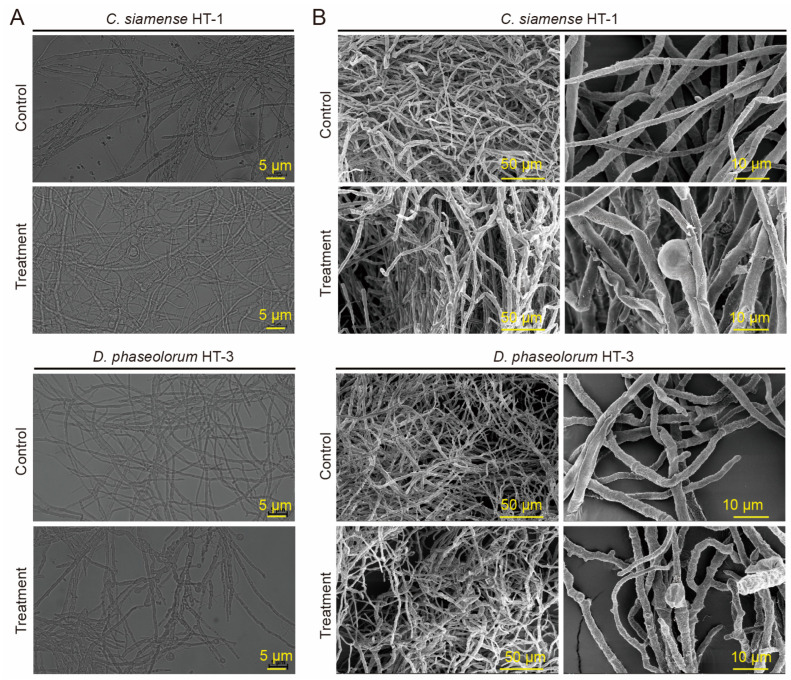
Effect of fermentation supernatant of strain GS2Y on the hyphal morphology of tea pathogenic fungi. (**A**) A fluorescence microscope was used to observe the hyphal morphology of *C. siamense* HT-1 and *D. phaseolorum* HT-3, which were co-cultured with 10% fermentation supernatant (FS) of strain GS2Y for 48 h. The bars represent the actual sizes. (**B**) Scanning electron microscopy (SEM) was used to observe the hyphal morphology of *C. siamense* HT-1 and *D. phaseolorum* HT-3 treated with 10% fermentation supernatant (FS) of strain GS2Y for 48 h. The bars represent the actual sizes.

To evaluate the inhibitory effect of strain GS2Y on the growth of pathogenic fungi affecting tea plants, a co-culture assay was performed using both the tea pathogenic fungi and the fermentation supernatant. The hyphal developmental morphology of the fungi was observed using a fluorescence microscope. The results indicated that the hyphal morphology of *C. siamense* HT-1 in the treated group exhibited notable enlargement, disordered growth, and swelling at the tip of the mycelium, whereas the hyphae of the control group remained intact and displayed normal radial growth (Figure 5A). Similarly, for *D. phaseolorum* HT-3, the hyphal morphology was comparable to that of *C. siamense* HT-1, with a more obvious swelling observed at the tips of the hyphae in the treatment group (Figure 5A). Subsequently, the hyphal morphology of the samples treated with 10% fermentation supernatant was further observed using a scanning electron microscope (SEM). The results showed that the hyphal morphology of *C. siamense* HT-1 treated with fermentation supernatant exhibited significant swelling and deformation, and some hyphae cell walls appeared rough and shriveled. In contrast, the hyphae in the control group appeared smooth, intact, and well defined (Figure 5B). Similarly, *D. phaseolorum* HT-3 treated with fermentation supernatant demonstrated a hyphal morphology comparable to that of *C. siamense* HT-1 (Figure 5B). These results indicate that strain GS2Y directly inhibits tea pathogenic fungi by disrupting their cellular structure.

### 3.6. Biocontrol Potential of Strain GS2Y Against Tea Pathogenic Fungi

**Figure 6:**
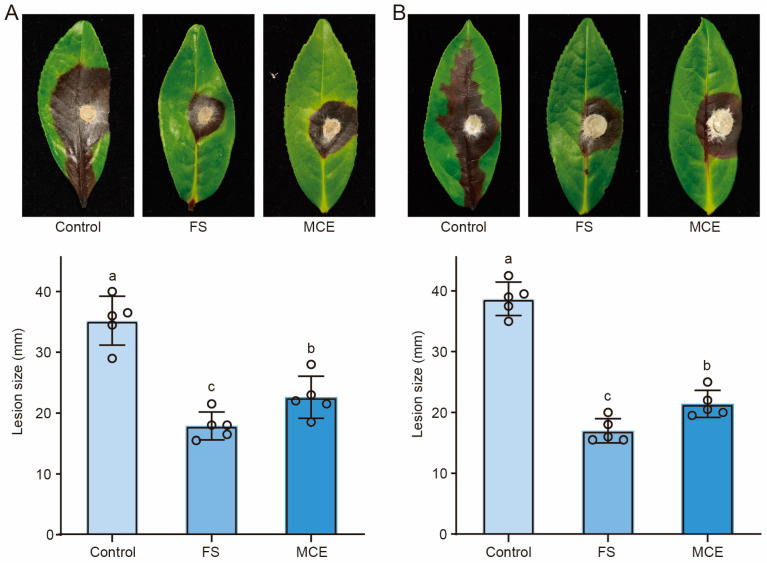
Biocontrol potential of strain GS2Y in tea plants. Fresh tea leaves were used for pathogenicity tests. Leaves were uniformly sprayed with either a 10% fermentation supernatant or a metabolic crude extract at a final concentration of 1.25 mg mL^−1^, with sterilized water serving as a control. Each leaf was punctured at the midpoint with a fine needle and placed in a sterile Petri dish containing two pieces of moist filter paper. A single piece of hyphae from either *C. siamense* HT-1 (**A**) or *D. phaseolorum* HT-3 (**B**) was then placed onto the leaf, ensuring contact between the hyphal side and the leaf surface. The plates were incubated in an illuminated chamber at 25 °C for 7 days. Representative leaves were photographed, and lesion sizes were measured. Each treatment group consisted of a minimum of five leaves. The circles represent the data points in the graph. The letters (a to c) above the columns indicate significant differences at *p* < 0.05, as determined using an unpaired two-tailed Student’s *t*-test analysis.

To explore the potential protective effects of the fermentation supernatant and metabolic crude extract derived from strain GS2Y against pathogenic fungal infections in tea plants, pathogenicity assays were performed on freshly harvested tea leaves. The experimental setup involved the application of 10% fermentation supernatant and 1.25 mg mL^−1^ metabolic crude extract, both of which were uniformly sprayed onto the fresh tea leaves, while sterilized water was utilized as a control. The results demonstrated that the fermentation supernatant significantly inhibited the infections caused by *C. siamense* HT-1 and *D. phaseolorum* HT-3 in the tea leaves, in contrast to the control group (Figure 6A,B). Furthermore, the metabolic crude extract also exhibited significant inhibitory effects on the infections induced by *C. siamense* HT-1 and *D. phaseolorum* HT-3, albeit with a weaker antifungal capacity compared to that of the fermentation supernatant (Figure 6A,B). In conclusion, we propose that strain GS2Y may serve as a potential biocontrol agent against fungal pathogens in tea plants.

## 4. Discussion

Tea plants cultivated in monoculture plantations in humid climates are susceptible to various biotic stresses (such as bacterial, fungal, and viral diseases), which adversely affect their growth and development, leading to significant global losses in tea yield and quality [10,61]. In our previous study, 49 rhizosphere soil samples were collected from both healthy and poorly managed tea plantations in Heshan, Southern China, to investigate soil fungal diversity [51]. In this study, three distinct fungal strains were isolated and identified from symptomatic leaves, *C. siamense* HT-1, *D. phaseolorum* HT-3, and *F. fujikuroi* HT-4 (Figure 1B), and their pathogenicity to tea trees was confirmed using Koch’s postulates. The pathogen *C. siamense* causes tea anthracnose, resulting in significant economic losses for the Chinese tea industry [62]. *D. phaseolorum* is known to cause stem canker on soybean and is also a destructive disease of rooibos tea (*Aspalathus linearis*) [52,63]. Here, the pathogenicity of *D. phaseolorum* was first isolated and verified in tea plants in China (Figure 1B). The genus *Fusarium* comprises a group of soil-borne fungi known to cause wilt, leaf spot, collar canker, and dieback in tea plants, with *F. fujikuroi* being the first reported fungus to cause tea wilt in Zhejiang Province, China [53]. The plant-pathogenic fungus *Pseudopestalotiopsis theae* causes tea gray blight, which is one of the most serious foliar diseases affecting tea trees [54]. Although *P. theae* was not isolated in this study, the genus *Pseudopestalotiopsis* remains one of the core taxa in the Heshan tea plantation, with a relative abundance of up to 4% in the tea plantation in Shuanghe town (Figure S2). Previous reports have indicated that *Alternaria longipes* and *Clonostachys rosea* can cause leaf spot disease in tea plants in China [55,56]. These results indicate that there might be a potential fungal disease risk in Heshan’s tea plantations.

Beneficial microorganisms are extensively employed in agriculture for controlling plant pathogens. However, the limited availability of efficacy and safety data has hindered the full utilization of promising biocontrol agents [64,65]. The search for new microorganisms as biocontrol agents is gaining attention from researchers. In this study, we identified nine cultivable bacterial strains from the rhizosphere soil and leaves of tea plants that exhibited antifungal activity against *D. phaseolorum* HT-3. These strains belonged to *Burkholderia*, *Streptomyces*, *Bacillus*, *Paenarthrobacter*, and *Sphingomonas* (Figure 2B). Previous studies have shown that these genera exhibited strong antifungal activity against plant pathogens [66,67,68]. *Streptomyces badius* gz-8 demonstrated significant antagonistic activity against *C. gloeosporioides*, achieving an inhibition rate of 72.5% [29]. *Bacillus velezensis* LJBV19 exhibited broad-spectrum antifungal activity and significantly inhibited the growth of *Magnaporthe oryzae* [69]. The microbial volatile compounds derived from *Paenarthrobacter ureafaciens* could inhibit the growth of phytopathogens, including *Lasiodiplodia theobromae* and *C. gloeosporioides* [68]. *Sphingomonas* asv20 exhibited strong suppression ability against *Diaporthe citri* under iron-deficient conditions [67].

In common with other biological control genera such as *Bacillus* and *Pseudomonas*, certain *Burkholderia* species can cause infections in humans, animals, and plants [70]. However, *Burkholderia* can also act as a biopesticide and promote plant growth [33,34]. Based on polyphasic taxonomic data, strain GS2Y represents a novel species within the genus *Burkholderia*. Our results demonstrated that this strain exhibited a wide spectrum of inhibitory activity against tea pathogenic fungi (Figure 4). The fermentation supernatant of this strain induced significant morphological changes in the mycelium, characterized by notable enlargement, disordered growth patterns, and tip swelling, with some hyphae cell walls appearing rough and shriveled (Figure 5B). These results suggest that strain GS2Y might disrupt the cellular structure of tea pathogenic fungi. A previous study has revealed that the secreted metabolites of *B. gladioli* KRS027 exhibited potent antifungal activity against *Botrytis cinerea*, causing hyphal deformation, fungal cell wall degradation, plasma membrane damage, organelle collapse, and cell content leakage [28]. Additionally, the results showed that GS2Y also could solubilize organic and inorganic phosphorus (Table S4), indicating its potential for controlling plant pathogenic fungi and promoting plant growth.

## 5. Conclusions

In summary, three tea pathogenic fungi were characterized, with *D. phaseolorum* being reported in tea plants in China for the first time in this study. Our results implied a potential disease risk in the tea plantations of Heshan, Southern China. Strain GS2Y, isolated from the rhizosphere soil of tea plants, was identified as a novel species of the genus *Burkholderia*, denoted *Burkholderia theae*, using morphological identification, phylogenetic analysis, and physiobiochemical characteristics. Strain GS2Y inhibited tea pathogenic fungi by significantly enlarging and disrupting the growth of mycelia and effectively prevented the infection of tea leaves by *C. siamense* HT-1 and *D. phaseolorum* HT-3. Due to strain GS2Y being derived from in situ soil, it might exhibit superior adaptability to the ecological environment of tea plantations. Therefore, *B. theae* GS2Y represents a potentially valuable resource for biocontrol agents. Future research endeavors will focus on the identification of the primary active constituents within the metabolic crude extracts derived from strain GS2Y, as well as the enhancement of the production processes for these principal active components. Detailed characterization of its antagonistic mechanisms will support the targeted development of biocontrol products for tea plants based on this strain.

## Data Availability

The original contributions presented in the study are included in the article/Supplementary Material; further inquiries can be directed to the corresponding authors. The GenBank/EMBL/DDBJ accession numbers for the 16S rRNA gene sequence and the whole genome of Burkholderia theae GS2Y are PQ219486 and JBCPYA000000000, respectively. The strain GS2Y has been deposited at the Guangdong Microbial Culture Collection Center (GDMCC) and Japan Collection of Microorganisms (JCM) (=GDMCC 64147 = JCM 36471).

## Supplementary Materials

The following supporting information can be downloaded at: https://www.mdpi.com/article/10.3390/cells13211768/s1, Figure S1. The colony morphology of tea pathogenic fungi isolated from the infected leaves was examined on PDA plates; Figure S2. Investigation of potential pathogenic fungi in tea plantation of Heshan of Southern China; Figure S3. Identification of strain GS2Y; Figure S4. The confrontation culture assays were performed to detect strain GS2Y against on *Phyllosticta captalensis* and *Gloeosporium musarum*; Figure S5. The minimum inhibitory concentrations (MICs) of metabolic crude extract (MCE) derived from strain GS2Y against *C. siamense* HT-1; Figure S6. The minimum inhibitory concentrations (MICs) of metabolic crude extract (MCE) derived from strain GS2Y against *D. phaseolorum* HT-3; Figure S7. The minimum inhibitory concentrations (MICs) of metabolic crude extract (MCE) derived from strain GS2Y against *F. fujikuroi* HT-4; Table S1. The cultivable bacteria isolated from the rhizosphere soil and leaves of tea plants in tea plantations located in Heshan City, Guangdong Province, China; Table S2. Genomic characteristic of strain GS2Y and their closely related strains of the genus *Burkholderia*; Table S3. The dDDH and ANI values between strains GS2Y and the related type strains of the genus *Burkholderia*; Table S4. Differential characteristics of strains GS2Y and their closely related strains of the genus *Burkholderia*; Table S5. Cellular fatty acid profiles of strain GS2Y and its closely related strains of the genus *Burkholderia*.
