# Supplementary material for: A Novel Strain Burkholderia theae GS2Y Exhibits Strong Biocontrol Potential Against Fungal Diseases in Tea Plants (Camellia sinensis)
Source: Cells. 2024 Oct 25;13(21):1768. doi: 10.3390/cells13211768 (PMC11545236; doi:10.3390/cells13211768)
Supplement: Supplementary file 1 [file cells-13-01768-s001.zip › cells-3228723-supplementary.pdf]

Article

A novel strain *Burkholderia theae* GS2Y exhibits strong biocontrol potential to fungal diseases in tea plants (*Camellia sinensis*)

Yijie Dong [1], Xing Wang [1], Guang-da Feng [1], Qing Yao [2] and Honghui Zhu [1,*]

[1] Key Laboratory of Agricultural Microbiomics and Precision Application (MARA), Guangdong Provincial Key Laboratory of Microbial Culture Collection and Application, Key Laboratory of Agricultural Microbiome (MARA), State Key Laboratory of Applied Microbiology Southern China, Guangdong Microbial Culture Collection Center (GDMCC), Institute of Microbiology, Guangdong Academy of Sciences, Guangzhou 510070, China; dongyj@gdim.cn (Y.D.); wangxingx7@163.com (X.W.); fenggd@gdim.cn (G-D.F.)

[2] Guangdong Province Key Laboratory of Microbial Signals and Disease Control, Guangdong Engineering Research Center for Litchi, College of Horticulture, South China Agricultural University, Guangzhou 510642, China; yaoqscau@scau.edu.cn

* Correspondence: zhuhh_gdim@163.com; Tel.: Tel: +86-20-87137669

Supplementary figures and legends

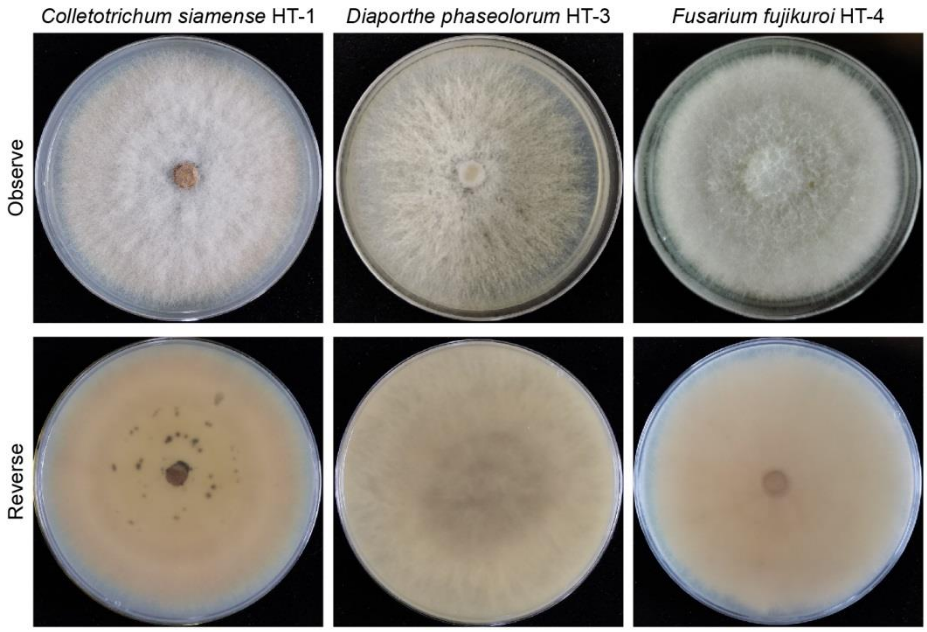

Figure S1. The colony morphology of tea pathogenic fungi isolated from the infected leaves was examined on PDA plate. *C. siamense* HT-1, *D. phaseolorum* HT-3, and *F. fujikuroi* HT-4 were incubated on the PAD plates at 30 °C for 5 days before photography was taken.

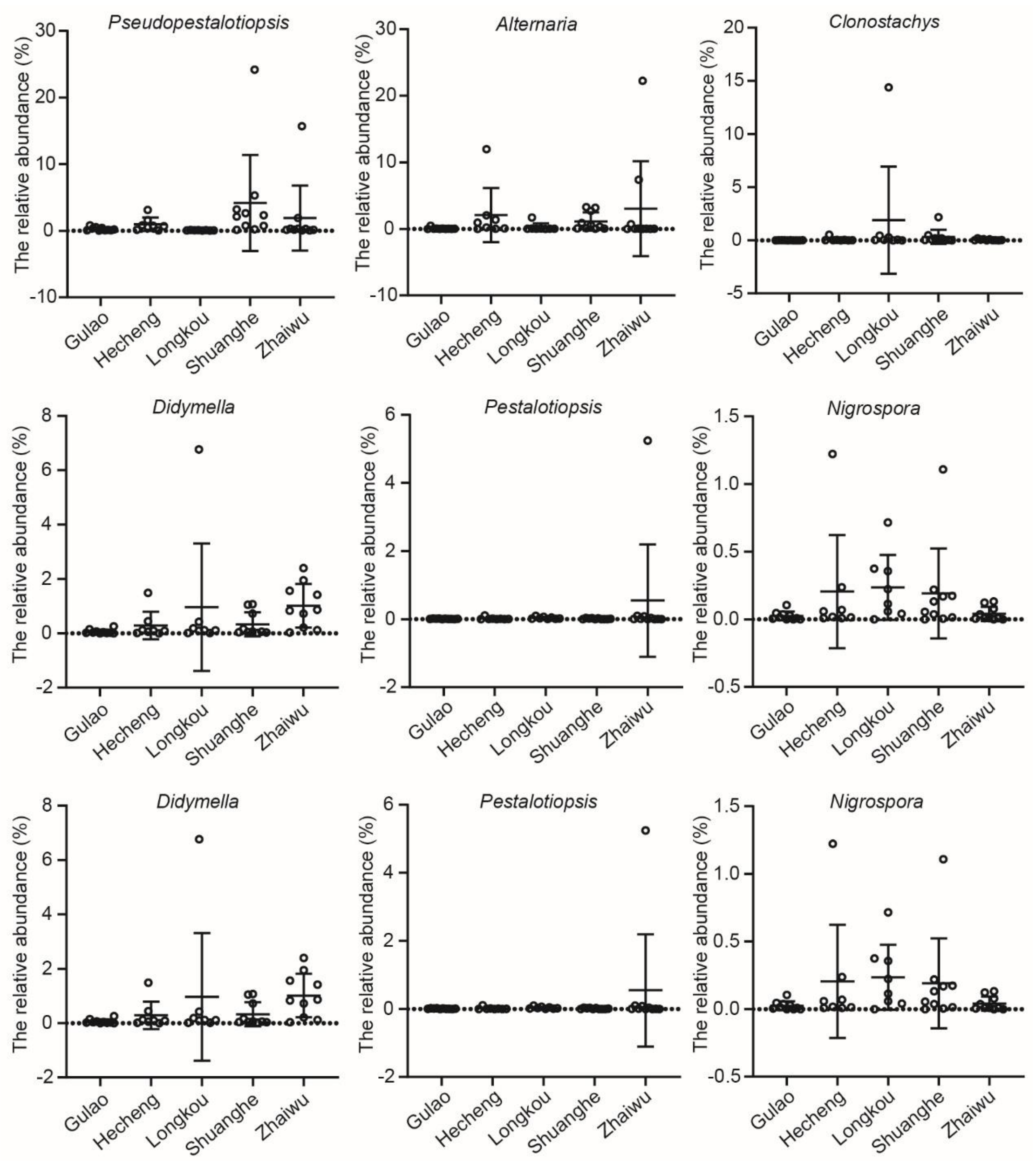

Figure S2. Investigation of potential pathogenic fungi in tea plantation of Heshan of southern China. The relative abundance of pathogenic fungi was detected based on the results of high-throughput sequencing. Gulao, Hecheng, Longkou, Shuanghe, and Zhaiwu represented the samples of tea plantation in Gulao town, Hecheng town, Longkou town, Shuanghe town and Zhaiwu town, respectively.

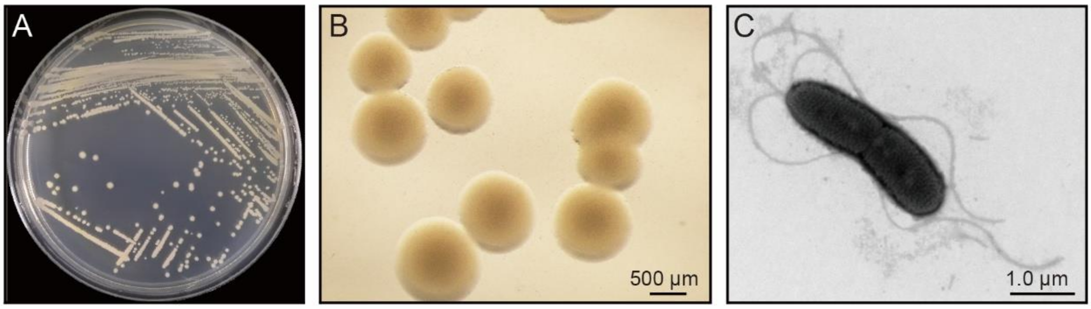

Figure S3. Identification of strain GS2Y. (A) The colony morphology growing on the LB plate was observed after culturing at 30 °C for 2 days prior to photography. (B) The colony morphology growing on the LB plate was observed at 30 °C for 2 days using a stereoscope. (C) The morphology of cells growing on the LB medium was observed at 30 °C for 48 h using the transmission electron microscopy (TEM).

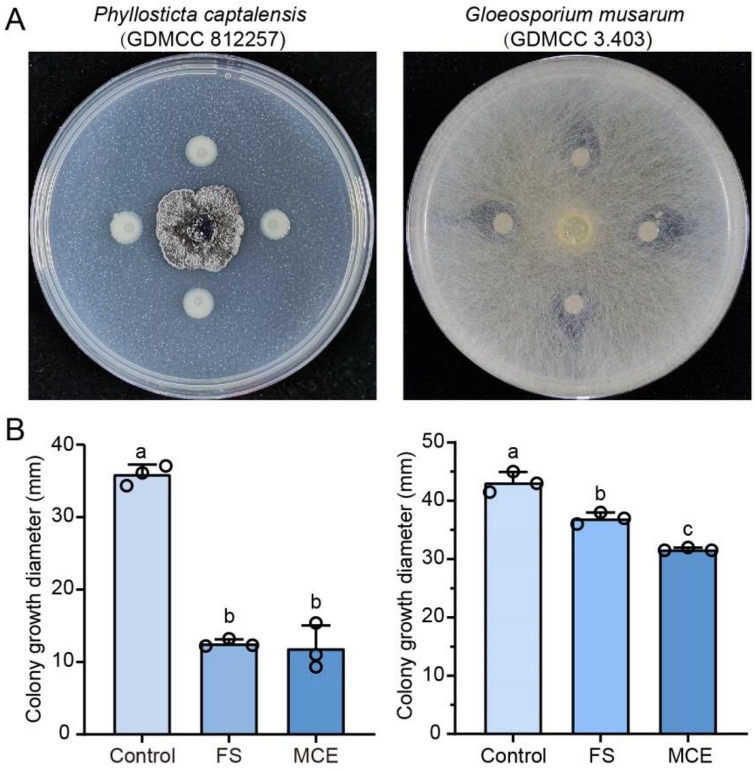

Figure S4. The confrontation culture assays were performed to detect strain GS2Y against on *Phyllosticta captalensis* and *Gloeosporium musarum*. (A) Mycelial discs of *P. captalensis* and *G. musarum* were placed in the center of PDA plates, and a suspension of strain GS2Y was incubated in a straight line around all four sides. The plates were incubated at 30°C for 5 days prior to photography. Each treatment included at least three biological replicates. (B) The growth inhibition tests were conducted to measure the antifungal activity of fermentation supernatant (FS) and metabolic crude extract (MCE) derived from strain GS2Y. A 6-mm diameter mycelial disc of *Phyllosticta captalensis* (GDMCC 812257) and *Gloeosporium musarum* (GDMCC 3.403) grown on PDA for five days was placed on the PDA plates containing 10% fermentation supernatant or a final concentration of 1.25 mg mL^{-1} of metabolic crude extract. The plates were incubated at 30 °C for five days, and the diameter of the growth inhibition zone was measured. The letters (a to c) above the columns indicate significant differences at $P<0.05$, as determined by an unpaired two-tailed Student’s t-test analysis.

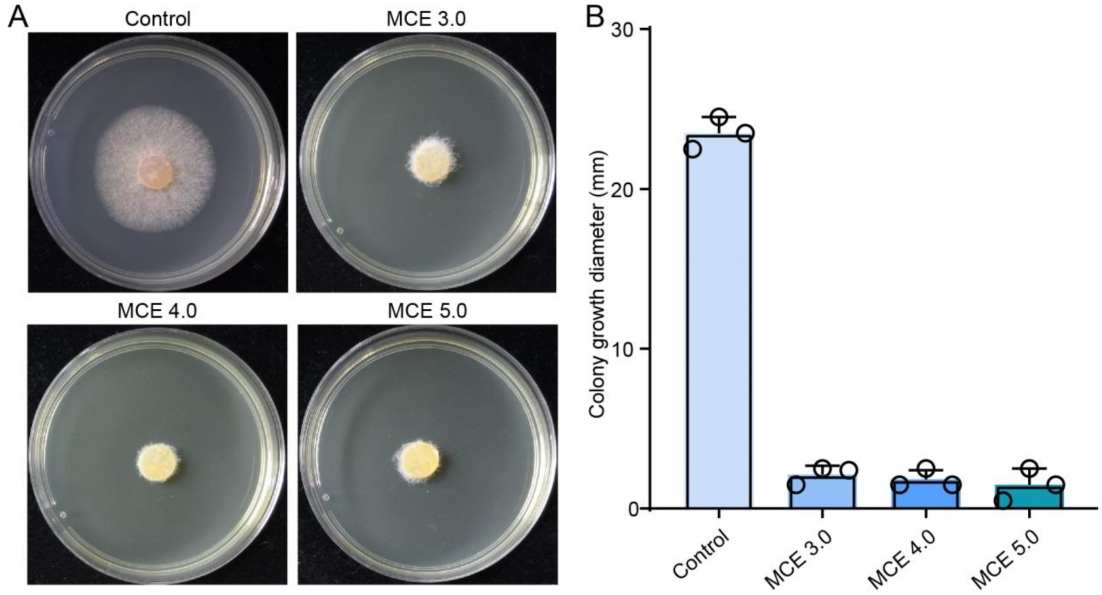

Figure S5 The minimum inhibitory concentrations (MICs) of metabolic crude extract (MCE) derived from strain GS2Y against *C. siamense* HT-1. Growth inhibition tests were conducted to assess the antifungal activity of MCE. A 6 mm diameter mycelial disc of *C. siamense* HT-1, cultivated on potato dextrose agar (PDA) for five days, was placed on PDA plates containing varying final concentrations of MCE. The plates were incubated at 30 °C for two days. (A) The colony morphology of *C. siamense* HT-1 was observed. (B) The diameter of the growth inhibition zone was measured. At least three biological replicates were performed.

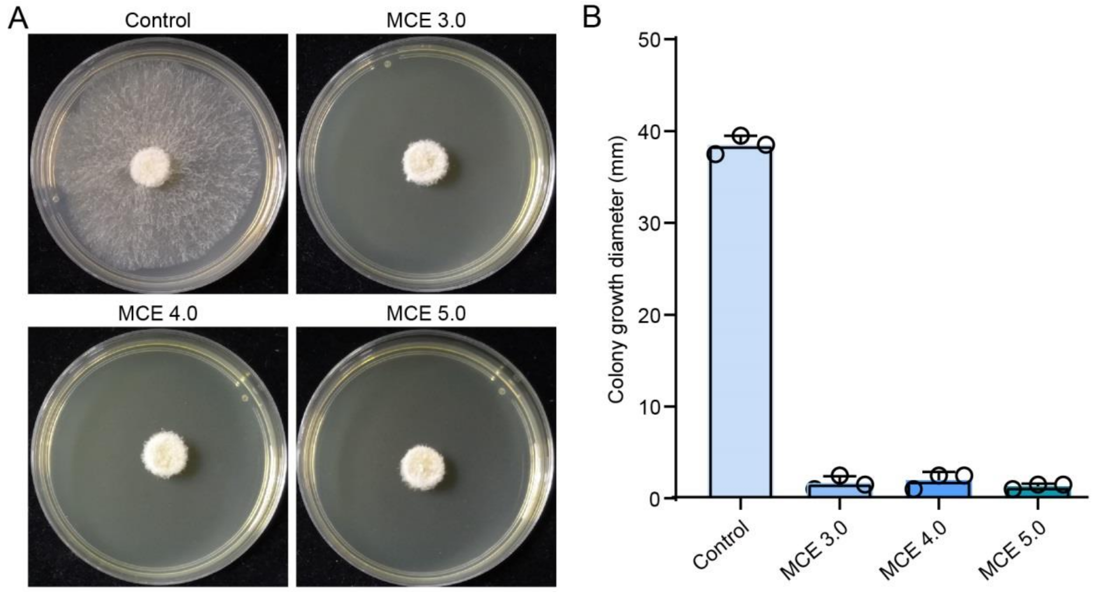

Figure S6 The minimum inhibitory concentrations (MICs) of metabolic crude extract (MCE) derived from strain GS2Y against *D. phaseolorum* HT-3. Growth inhibition tests were conducted to assess the antifungal activity of MCE. A 6 mm diameter mycelial disc of *D. phaseolorum* HT-3, cultivated on potato dextrose agar (PDA) for five days, was placed on PDA plates containing varying final concentrations of MCE. The plates were incubated at 30 °C for two days. (A) The colony morphology of *D. phaseolorum* HT-3 was observed. (B) The diameter of the growth inhibition zone was measured. At least three biological replicates were performed.

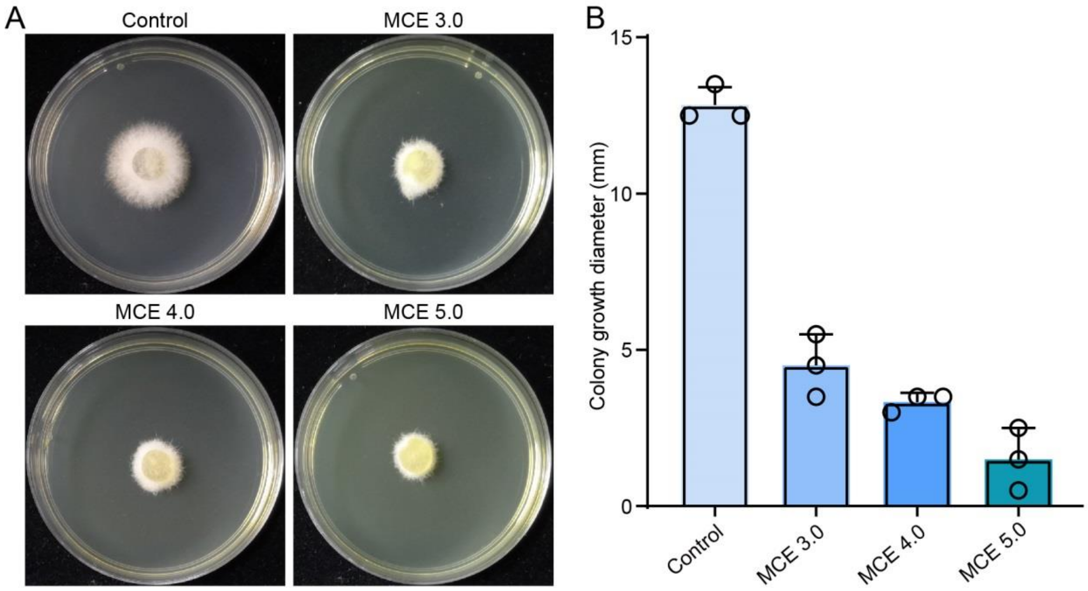

Figure S7 The minimum inhibitory concentrations (MICs) of metabolic crude extract (MCE) derived from strain GS2Y against *F. fujikuroi* HT-4. Growth inhibition tests were conducted to assess the antifungal activity of MCE. A 6 mm diameter mycelial disc of *F. fujikuroi* HT-4, cultivated on potato dextrose agar (PDA) for five days, was placed on PDA plates containing varying final concentrations of MCE. The plates were incubated at 30 °C for two days. (A) The colony morphology of *F. fujikuroi* HT-4 was observed. (B) The diameter of the growth inhibition zone was measured. At least three biological replicates were performed.

Supplementary Tables

Table S1. The cultivable bacteria isolated from the rhizosphere soil and leaves of tea plants in tea plantations located in Heshan City, Guangdong Province, China

Isolates	Isolated name	GDMCC numbers	Hit strain name	Similarity (%)
G1	*Pedobacter* sp.	GDMCC 809125	*Pedobacter terrae* DSM 17933	98.2
G10F	*Curtobacterium oceanosedimentum*	GDMCC 809126	*Curtobacterium oceanosedimentum* ATCC 31317	99.9
G12F	*Bacillus cereus*	GDMCC 809127	*Bacillus cereus* ATCC 14579	99.7
G2	*Pseudomonas glycinae*	GDMCC 809128	*Pseudomonas glycinae* MS586	99.9
G21	*Variovorax paradoxus*	GDMCC 809129	*Variovorax paradoxus* NBRC 15149	99.5
G22	*Microbacterium testaceum*	GDMCC 809130	*Microbacterium testaceum* NBRC 12675	100
G25	*Sphingomonas sanguinis*	GDMCC 809131	*Sphingomonas sanguinis* NBRC 13937	99.3
G31	*Arthrobacter celericrescens*	GDMCC 809132	*Arthrobacter celericrescens* NEAU-SA2	99.3
G34	*Agromyces* sp.	GDMCC 809133	*Agromyces binzhouensis* OAct353	98.6
G4	*Pantoea rodasii*	GDMCC 809134	*Pantoea rodasii* LMG 26273	98.7
G6F	*Pseudomonas azotoformans*	GDMCC 809135	*Pseudomonas azotoformans* DSM 18862	99.9
G8F	*Curtobacterium flaccumfaciens*	GDMCC 809136	*Curtobacterium flaccumfaciens* LMG 3645	99.8
G9	*Stenotrophomonas indicatrix*	GDMCC 809137	*Stenotrophomonas indicatrix* WS40	99.7
GS1	*Streptomyces resistomycificus*	GDMCC 809060	*Streptomyces resistomycificus* NRRL ISP-5133	98.9
GS10	*Bacillus paranthracis*	GDMCC 809061	*Bacillus paranthracis* Mn5	100
GS16	*Burkholderia ambifaria*	GDMCC 809062	*Burkholderia ambifaria* AMMD	99.7

GS1Y	*Arthrobacter terricola*	GDMCC 809063	*Arthrobacter terricola* JH1-1	99.8
GS2	*Streptomyces* sp.	GDMCC 809064	*Streptomyces ardesiacus* NRRL B-1773	98.4
GS23	*Chryseobacterium lactis*	GDMCC 809065	*Chryseobacterium lactis* NCTC 11390	98.8
GS28	*Arthrobacter ramosus*	GDMCC 809066	*Arthrobacter ramosus* CCM 1646	99.5
GS2Y	*Burkholderia contaminans*	GDMCC 809067	Burkholderia contaminans LMG 23361	99.8
GS30	*Paenarthrobacter nicotinovorans*	GDMCC 809068	*Paenarthrobacter nicotinovorans* DSM 420	100
GS34	*Paenibacillus* sp.	GDMCC 809069	*Paenibacillus amylolyticus* NBRC 15957	98.2
GS8	*Paraburkholderia acidiphila*	GDMCC 809070	*Paraburkholderia acidiphila* 7Q-K02	99.3
GS9	*Burkholderia aenigmatica*	GDMCC 809071	*Burkholderia aenigmatica* LMG 13014	99.9
H12	*Curtobacterium oceanosedimentum*	GDMCC 809138	*Curtobacterium oceanosedimentum* ATCC 31317	99.8
H14	*Microbacterium testaceum*	GDMCC 809139	*Microbacterium testaceum* NBRC 12675	100
H2	*Acidovorax oryzae*	GDMCC 809140	*Acidovorax oryzae* ATCC 19882	100
H21	*Bacillus cereus*	GDMCC 809141	*Bacillus cereus* ATCC 14579	100
H25	*Staphylococcus warneri*	GDMCC 809142	*Staphylococcus warneri* ATCC 27836	99.5
H29	*Chryseobacterium camelliae*	GDMCC 809143	*Chryseobacterium camelliae* THG C4-1	99.9
H31	*Stenotrophomonas indicatrix*	GDMCC 809144	*Stenotrophomonas indicatrix* WS40	99.7
H32	*Sphingomonas yabuuchiae*	GDMCC 809145	*Sphingomonas yabuuchiae* GTC 868	99.3
H33	*Pseudomonas parafulva*	GDMCC 809146	*Pseudomonas parafulva* NBRC 16636	99.9
H4F	*Agrobacterium cavarae*	GDMCC 809147	*Agrobacterium cavarae* RZME10	99.9
HS13	*Pseudomonas plecoglossicida*	GDMCC 809072	*Pseudomonas plecoglossicida* NBRC 103162	99.7

HS17	*Sinomonas* sp.	GDMCC 809073	*Sinomonas humi* MUSC 117	97.3
HS24	*Arthrobacter celericrescens*	GDMCC 809074	*Arthrobacter celericrescens* NEAU-SA2	99.1
HS26	*Paenibacillus oryzae*	GDMCC 809075	*Paenibacillus oryzae* 1DrF-4	98.7
HS28	*Arthrobacter pascens*	GDMCC 809076	*Arthrobacter pascens* DSM 20545	99.4
HS34	*Pseudarthrobacter siccitolerans*	GDMCC 809077	*Pseudarthrobacter siccitolerans* 4J27	99.3
HS37	*Microbacterium* sp.	GDMCC 809078	*Microbacterium lushaniae* L-031	97.9
HS39	*Bacillus altitudinis*	GDMCC 809079	*Bacillus altitudinis* 41KF2b	100
HS4	*Paenarthrobacter nicotinovorans*	GDMCC 809080	*Paenarthrobacter nicotinovorans* DSM 420	99.6
HS43	*Arthrobacter* sp.	GDMCC 809081	*Arthrobacter pokkalii* P3B162	98.6
HS5	*Agromyces italicus*	GDMCC 809082	*Agromyces italicus* DSM 16388	99.4
HS6	*Arthrobacter globiformis*	GDMCC 809083	*Arthrobacter globiformis* NBRC 12137	98.8
HS7	*Arthrobacter gyeryongensis*	GDMCC 809084	*Arthrobacter gyeryongensis* DCY72	99
HS8	*Stenotrophomonas maltophilia*	GDMCC 809085	*Stenotrophomonas maltophilia* MTCC 434	99.9
HS9	*Sinomonas* sp.	GDMCC 809086	*Sinomonas soli* CW 59	98.1
L11	*Acidovorax oryzae*	GDMCC 809148	*Acidovorax oryzae* ATCC 19882	99.9
L11F	*Bacillus cereus*	GDMCC 809149	*Bacillus cereus* ATCC 14579	100
L16	*Microbacterium testaceum*	GDMCC 809150	*Microbacterium testaceum* NBRC 12675	99.9
L20	*Herbaspirillum aquaticum*	GDMCC 809151	*Herbaspirillum aquaticum* IEH 4430	99.8
L26	*Burkholderia contaminans*	GDMCC 809152	*Burkholderia contaminans* LMG 23361	100
L29	*Bacillus altitudinis*	GDMCC 809153	*Bacillus altitudinis* 41KF2b	99.9

L3F	*Xanthomonas sacchari*	GDMCC 809154	*Xanthomonas sacchari* LMG 471	99.6
L4	*Sphingomonas yabuuchiae*	GDMCC 809155	*Sphingomonas yabuuchiae* GTC 868	99.3
L40	*Pseudomonas azotoformans*	GDMCC 809156	*Pseudomonas azotoformans* DSM 18862	99.9
L5	*Curtobacterium citreum*	GDMCC 809157	*Curtobacterium citreum* DSM 20528	99.9
L6	*Luteibacter anthropi*	GDMCC 809158	*Luteibacter anthropi* CCUG 25036	98.9
L7	*Herbaspirillum seropedicae*	GDMCC 809159	*Herbaspirillum seropedicae* Z67	99.7
L7F	*Curtobacterium oceanosedimentum*	GDMCC 809160	*Curtobacterium oceanosedimentum* ATCC 31317	99.1
LS10	*Bacillus tropicus*	GDMCC 809087	*Bacillus tropicus* N24	100
LS11	*Staphylococcus epidermidis*	GDMCC 809088	*Staphylococcus epidermidis* NCTC 11047	100
LS13	*Paraburkholderia acidiphila*	GDMCC 809089	*Paraburkholderia acidiphila* 7Q-K02	99.2
LS14	*Bacillus albus*	GDMCC 809090	*Bacillus albus* N35-10-2	100
LS18	*Ralstonia mannitolilytica*	GDMCC 809091	*Ralstonia mannitolilytica* LMG 6866	99.2
LS22	*Paenarthrobacter nitroguajacolicus*	GDMCC 809092	*Paenarthrobacter nitroguajacolicus* G2-1	98.9
LS27	*Arthrobacter woluwensis*	GDMCC 809093	*Arthrobacter woluwensis* NBRC 107840	99.0
LS28	*Brevibacillus choshinensis*	GDMCC 809094	*Brevibacillus choshinensis* DSM 8552	99.1
LS32	*Sinomonas* sp.	GDMCC 809095	*Sinomonas susongensis* A31	98.5
LS5	*Lysinibacillus fusiformis*	GDMCC 809096	*Lysinibacillus fusiformis* NBRC 15717	99.9
LS9Y	*Bacillus velezensis*	GDMCC 809097	*Bacillus velezensis* CR-502	99.9
S11	*Acidovorax oryzae*	GDMCC 809161	*Acidovorax oryzae* ATCC 19882	99.9
S26	*Microbacterium testaceum*	GDMCC 809162	*Microbacterium testaceum* NBRC 12675	100

S27	*Sphingomonas sanguinis*	GDMCC 809163	*Sphingomonas sanguinis* NBRC 13937	99.1
S33	*Enterobacter wuhouensis*	GDMCC 809164	*Enterobacter wuhouensis* WCHEW120002	99.7
S36	*Chryseobacterium camelliae*	GDMCC 809165	*Chryseobacterium camelliae* THG C4-1	99.9
S6F	*Curtobacterium oceanosedimentum*	GDMCC 809166	*Curtobacterium oceanosedimentum* ATCC 31317	99.6
S8	*Agromyces* sp.	GDMCC 809167	*Agromyces binzhouensis* OAct353	98.5
SS11	*Sinomonas* sp.	GDMCC 809098	*Sinomonas susongensis* A31	98.3
SS12	*Amycolatopsis echigonensis*	GDMCC 809099	*Amycolatopsis echigonensis* LC2	99.1
SS15	*Sinomonas atrocyanea*	GDMCC 809100	*Sinomonas atrocyanea* KCTC 3377	99.6
SS18	*Rhodococcus hoagii*	GDMCC 809101	*Rhodococcus hoagii* DSM 20295	99.9
SS19	*Arthrobacter alkaliphilus*	GDMCC 809102	*Arthrobacter alkaliphilus* LC6	99.4
SS25	*Streptomyces albospinus*	GDMCC 809103	*Streptomyces albospinus* NBRC 13846	99.6
SS26	*Dyella marensis*	GDMCC 809104	*Dyella marensis* CS5-B2	100
SS28	*Rhodococcus agglutinans*	GDMCC 809105	*Rhodococcus agglutinans* CCTCC AB2014297	99.1
SS3	*Brevibacillus choshinensis*	GDMCC 809168	*Brevibacillus choshinensis* DSM 8552	99.3
SS30	*Streptomyces gelaticus*	GDMCC 809106	*Streptomyces gelaticus* NRRL B-2928	99.4
SS7	*Stenotrophomonas maltophilia*	GDMCC 809107	*Stenotrophomonas maltophilia* MTCC 434	99.4
W12	*Variovorax paradoxus*	GDMCC 809169	*Variovorax paradoxus* NBRC 15149	99.3
W15	*Pedobacter terrae*	GDMCC 809170	*Pedobacter terrae* DSM 17933	98.8
W21	*Acinetobacter johnsonii*	GDMCC 809171	*Acinetobacter johnsonii* CIP 64.6	99.4
W24	*Chryseobacterium hagamense*	GDMCC 809172	*Chryseobacterium hagamense* RHA2-9	99.1

W6F	*Pseudomonas azotoformans*	GDMCC 809173	*Pseudomonas azotoformans* DSM 18862	99.9
W8	*Microbacterium testaceum*	GDMCC 809174	*Microbacterium testaceum* NBRC 12675	99.7
W8F	*Agrobacterium larrymoorei*	GDMCC 809175	*Agrobacterium larrymoorei* ATCC 51759	99.5
WS1	*Arthrobacter bambusae*	GDMCC 809108	*Arthrobacter bambusae* GM18	99.3
WS11	*Sinomonas* sp.	GDMCC 809109	*Sinomonas humi* MUSC 117	98.3
WS12	*Terrabacter koreensis*	GDMCC 809110	*Terrabacter koreensis* THG-e54	99.5
WS13	*Burkholderia arboris*	GDMCC 809111	*Burkholderia arboris* R-24201	99.8
WS16	*Sinomonas atrocyanea*	GDMCC 809112	*Sinomonas atrocyanea* KCTC 3377	99.7
WS18	*Lysinibacillus xylanilyticus*	GDMCC 809113	*Lysinibacillus xylanilyticus* DSM 23493	99.2
WS2	*Streptomyces crystallinus*	GDMCC 809114	*Streptomyces crystallinus* NBRC 15401	99.6
WS23	*Ralstonia insidiosa*	GDMCC 809115	*Ralstonia insidiosa* AU2944	99.0
WS28	*Methylobacterium oryzae*	GDMCC 809116	*Methylobacterium oryzae* CBMB20	99.7
WS3	*Streptomyces tsukubensis*	GDMCC 809117	*Streptomyces tsukubensis* NRRL 18488	99.9
WS30	*Streptomyces setonii*	GDMCC 809118	*Streptomyces setonii* NRRL ISP-5322	99.8
WS31	*Paenarthrobacter nicotinovorans*	GDMCC 809119	*Paenarthrobacter nicotinovorans* DSM 420	100
WS32	*Arthrobacter terricola*	GDMCC 809120	*Arthrobacter terricola* JH1-1	99.8
WS35	*Buttiauxella agrestis*	GDMCC 809121	*Buttiauxella agrestis* ATCC 33320	98.8
WS5	*Brevibacterium frigoritolerans*	GDMCC 809122	*Brevibacterium frigoritolerans* DSM 8801	99.9
WS6	*Bacillus tequilensis*	GDMCC 809123	*Bacillus tequilensis* KCTC 13622	99.9
WS8	*Paenibacillus* sp.	GDMCC 809124	*Paenibacillus daejeonensis* AP-20	98.1

Table S2. Genomic characteristic of strain GS2Y and their closely related strains of the genus *Burkholderia*

Genomic features	1	2	3
Size (Mbp)	8.6	8.0	8.5
Contigs numbers	44	4	3
DNA G+C content (%)	66.5	66.5	66.5
Contigs N50 (Kbp)	376.7	3200	3300
Total gene numbers	7724	7223	7849
CDS numbers	7547	6997	7600
The number of rRNA genes	3	19	18
The number of tRNA genes	56	68	69
Completeness (%)	100	100	98.75
Contamination (%)	1.24	0.88	0.49

Strains: 1, GS2Y; 2, *Burkholderia pyrrocinia* DSM 10685; 3, *Burkholderia stabilis* ATCC BAA-67.

Table S3. The dDDH and ANI values between strains GS2Y and the related type strains of the genus *Burkholderia*

Strain	ANI(%)	dDDH(%)
Burkholderia pyrrocinia DSM 10685	93.9	52.2
Burkholderia catarinensis DSM 103188	93.3	49.5
Burkholderia stabilis ATCC BAA-67	93.1	48.8
Burkholderia cepacia ATCC 25416	92.5	45.4
Burkholderia puraquae LMG 29660	92.5	45.8
Burkholderia seminalis LMG 24067	92.4	45.4
Burkholderia reimsis BE51	92.4	45.2
Burkholderia lata 383	92.4	45.3
Burkholderia cenocepacia LMG 16656	92.3	44.6
Burkholderia aenigmatica LMG 13014	92.3	45.2
Burkholderia metallica LMG 24068	92.2	44.2
Burkholderia contaminans LMG 23361	92.1	44.1
Burkholderia paludis MSh1	91.8	43.5
Burkholderia diffusa DSM 23434	90.9	39.9
Burkholderia anthina LMG 20980	90.9	39.8
Burkholderia ambifaria AMMD	90.8	39.9
Burkholderia territorii LMG 28158	90.6	38.7

Burkholderia latens CCUG 54555	90.2	38.8
Burkholderia vietnamiensis LMG 10929	89.2	35.8
Burkholderia stagnalis LMG 28156	90.0	33.7

Table S4. Differential characteristics of strains GS2Y and their closely related strains of the genus *Burkholderia*

**Characteristics**	**1**	**2**	**3**
Isolation source	Soil	Soil	Sputum
Growth for			
Temperature range (°C) (optimum)	4–42 (28–30)	4–42 (28–30)	4–42 (28–30)
pH range (optimum)	3.0–9.0 (6.0–8.0)	3.0–9.0 (6.0–8.0)	3.0–9.0 (6.0–8.0)
NaCl concentration (%) (optimum)	0–3.0 (0–0.5)	0–3.0 (0–0.5)	0–3.0 (0–0.5)
Hydrolysis of:			
Cellulose	+	+	+
Tween 20	+	+	+
Tween 40	+	+	+
Tween 60	+	+	+
Organic phosphorus	+	+	+
Inorganic phosphorus	+	+	+
API 20NE			
Reduction of nitrate	+	-	-
Indole production	-	-	-
D-glucose fermentation	+	+	+
Arginine dihydrolase	+	+	+
Urease	+	+	+
β-glucosidase	+	+	+
Gelatin hydrolysis	+	+	+
β-galactosidase	+	+	-
Utilization of:			
D-glucose	+	+	+
L-arabinose	+	+	+
D-mannose	+	+	+
D-mannitol	+	+	+

N-acetyl-glucosamine	+	+	+
D-maltose	+	-	-
Potassium gluconate	+	+	+
Capric acid	+	+	+
Adipic acid	+	+	+
Malic acid	+	+	+
Trisodium citrate	+	+	+
Phenylacetic acid	+	+	+

Strains: 1, GS2Y; 2, *Burkholderia pyrrocinia* DSM 10685; 3, *Burkholderia stabilis* ATCC BAA-67.

Table S5. Cellular fatty acid profiles of strain GS2Y and its closely related strains of the genus *Burkholderia*

**Fatty acids**	**1**	**2**	**3**
$C_{12:00}$	3.41	3.17	0.32
$C_{14:00}$	0.94	0.97	4.84
**$C_{16:00}$**	**23.95**	**24.78**	**25.26**
$C_{18:00}$	0.88	1.29	1.05
$C_{16:0}$ 2OH	2.66	1.62	1.26
$C_{16:0}$ 3OH	4.31	3.93	4.4
$C_{16:1}$ 2OH	1.47	1.07	0.87
**$C_{17:0}$ cyclo**	**21.53**	**24.05**	**20.56**
$C_{18:1}$ 2OH	2.48	1.8	nd
**$C_{19:0}$ cyclo *w8c***	**22.18**	**14.27**	**15.06**
Summed Feature 2	3.77	3.85	5.21
Summed Feature 3	1.13	2.52	6.69
**Summed Feature 8**	7.86	**13.69**	**11.7**

Strains: 1, GS2Y; 2, *Burkholderia pyrrocinia* DSM 10685; 3, *Burkholderia stabilis* ATCC BAA-67.

Data on the fatty acids were obtained from this study. Less than 1.0% and/or the absence of fatty acids for all strains are not shown. Values were percentages of total fatty acids. The predominant cellular fatty acids for all strains (> 10%) are in bold. nd, not detected. summed feature 2, $C_{12:0}$ aldehyde?; summed feature 3, $C_{16:1}$ *w7c* and/or $C_{16:1}$ *w6c*; summed feature 8, $C_{18:1}$ *w7c*.
